# The significance of MRI‐detected lateral lymph node metastasis in rectal cancer on local recurrence and survival—A nationwide cohort study

**DOI:** 10.1111/codi.70377

**Published:** 2026-01-26

**Authors:** Erik Agger, Cecilia Dahlbäck, Cedric Delorme, Pamela Buchwald

**Affiliations:** ^1^ Faculty of Medicine Lund University Lund Sweden; ^2^ Department of Surgery Skåne University Hospital Malmö Sweden

**Keywords:** lateral lymph nodes, local recurrence, overall survival, rectal cancer

## Abstract

**Aim:**

This retrospective observational cohort study aimed to investigate the prevalence of suspected lateral lymph node metastases (LLNM), as diagnosed by magnetic resonance imaging (MRI), and its impact on local recurrence (LR) and overall survival (OS). Patients who underwent dissection of suspected lymph nodes were evaluated in subgroup analysis.

**Method:**

Patients diagnosed with rectal cancer in Sweden between 2017 and 2021 were identified through the Swedish colorectal cancer registry and grouped by MRI findings. Main outcome measures were LR at 3 years and OS at 3 and 5 years.

**Results:**

In all, 2779 patients were analysed. Frequency of lateral lymph node (LLN) enlargement on MRI was 9.4%. In univariable analysis, the risk of LR at 3 years was increased in LLN‐positive patients, HR 2.79 (CI 1.55–5.03). In multivariable analysis, adjusted for patient, tumour and neoadjuvant treatment factors, this difference remained (HR 1.97 (CI 1.04–3.73)). No difference in 3‐year OS was observed. At 5 years, univariable analysis indicated increased mortality in LLN‐positive patients (HR 1.64 (CI 1.24–2.17)), but not in multivariable analysis.

Among LLN‐positive patients, those who had undergone LLN dissection (LLND) had an LR rate of 12.5% and increased risk at 3 years in univariable analysis (HR 3.73 (CI 1.93–10.76)). However, in multivariable analysis, there was no difference in LR risk or OS.

**Conclusion:**

MRI‐detected LLN positivity is associated with a higher risk of LR and correlates with other adverse prognostic factors. The effect of LLND on LR remains unclear. Further research is needed to optimize treatment strategies for patients with suspected LLNM.

**What does this paper add to the literature?**

This study underscores the prognostic role of radiologically diagnosed lateral lymph node metastasis in predicting local recurrence but not overall survival in rectal cancer. The effect of lateral lymph node dissection remains elusive, highlighting the necessity of future research to refine treatment strategies.

## INTRODUCTION

Management of lateral lymph node metastases (LLNM) in rectal cancer continues to spark discussion within the colorectal community [1, 2, 3, 4].

Presence of LLNM is associated with both increased risk of local recurrence (LR) and reduced survival rates [5, 6, 7]. Previous studies indicate that patients with suspected LLNM may benefit from targeted treatment strategies of the lateral lymph node compartments, which is the most common location for LR [8, 9, 10, 11]. Improving management of suspected LLNM may play a crucial role in preventing LR in rectal cancer [10, 12].

Treatment strategies for LLNM may involve (chemo)radiotherapy and/or lateral lymph node dissection (LLND), but an international consensus on how to manage LLNM is still lacking [1, 9, 13, 14, 15, 16].

The lack of consensus on how lateral lymph nodes (LLN) should be managed in rectal cancer treatment is based on conflicting views on this aspect of advanced disease. LLNM may be defined as a sign of local disease and prophylactic LLND as the primary treatment in patients with rectal tumours below the peritoneal reflection if the tumour extends beyond the muscularis propria [17]. LLNM may also be seen as a sign of more disseminated disease, and suspected LLN involvement should therefore be treated primarily with an oncological approach. In Sweden, national guidelines stipulated a treatment strategy involving neoadjuvant chemoradiotherapy (CRT) followed by re‐evaluation. For patients with persistent LLN enlargement after neoadjuvant therapy, LLND should be considered to mitigate the risk of LR [18]. This strategy is in line with guidelines published by the European Society of Medical Oncology (ESMO), namely that LLN should only be removed if they have persisting malignant features after CRT [19].

As management for patients with suspected LLNM in rectal cancer diverges, rigorous evaluation and further research is warranted to conclude an optimal treatment strategy.

### Aim

The aim of the study was to investigate the prevalence of MRI‐detected LLN positivity in rectal cancer and if LLN positivity is associated with LR and overall survival (OS) in a nationwide cohort. Secondary aims were to evaluate how LLND affected LR and overall survival.

## METHOD

### Study population

Patients diagnosed with rectal cancer (ICD‐10; C20.9) between 1 January 2017 and 31 December 2021, who had undergone rectal cancer surgery and who had an accessible pre‐treatment radiology assessment, were identified through the Swedish Colorectal Cancer Registry (SCRCR), a comprehensive national database covering 98.8% of rectal cancer patients with prospectively registered data [20]. Rectal cancer was defined as an adenocarcinoma with the lower border located ≤15 cm from the anal verge measured with rigid sigmoidoscopy. MRI‐based measurement was not used for height estimation during the study period. Patients with stage 1–4 rectal cancer treated with abdominal rectal resection with or without LLND were included for analysis. Stage 4 patients were included in the study cohort with the aim of providing a comprehensive presentation of patients with suspected LLNM as distant metastases are not uncommon in patients with advanced pelvic disease. Exclusion criteria were non‐radical resection and early recurrence (within 90 days).

In the registry, LLN positivity was recorded based on the pre‐treatment MRI using size criteria before neoadjuvant therapy. However, specific measurement of node size or other malignant features were not prospectively registered and could not be analysed. According to Swedish guidelines, LLN with a short axis of >7 mm or a long axis of >10 mm indicates a high risk of LLNM [18, 21]. The definition of LLN‐positive/negative in the current study refers exclusively to radiologically suspected nodes, not histologically verified metastases.

Consistent with treatment guideline, LLND was performed in patients with persistent cross‐section LLN size of ≥5 mm upon re‐evaluation. In Sweden, enlarged lymph nodes along the internal and obturator arteries are classified as N‐disease, whereas positive nodes along the external and common iliac arteries are considered metastatic disease [18]. LLND was considered performed when indicated and properly documented in the free‐text section describing extended resection within the SCRCR. The free‐text section captures all information related to surgical procedures beyond TME; the entries are neither validated nor standardized. During the inclusion period, LLND surgery was not formally centralized nationally and data reflecting experience in LLND and case volume was not available.

LR was defined as intraluminal recurrence or peritoneal tumour growth below the promontory, or as recurrence in local lymph nodes, diagnosed >90 days after surgical resection.

The SCRCR provided data on diagnosed LR with continuous updates on the date of LR‐diagnosis throughout the follow‐up period after surgery. Additionally, the registry conducts follow‐up medical record reviews at 3 and 5 years after surgical resection. While individual follow‐up protocols may vary, they are expected to align with registry reporting intervals. Guideline‐mandated follow‐up includes radiological imaging for recurrence detection at one and 3 years after surgery. The date of death was retrieved from the Cause of Death Registry as of 8 June 2023.

Histopathology reports of a regional subgroup of patients who had undergone LLND were retrieved to examine the prevalence of LLNM in the resected specimens.

### Statistical analysis

The prevalence of suspected LLNM on MRI was evaluated. Comparative analyses between LLN‐positive and LLN‐negative groups were conducted, examining demographic variables, preoperative and postoperative TNM staging, presence of positive lymph nodes in other locations, as well as neoadjuvant and surgical treatment strategies.

Categorical variables were expressed as counts and percentages, while continuous numerical variables were presented as means with standard deviations. Pearson's *χ* ^2^‐test was used for categorical comparisons, with Fisher's exact test applied when more than 20% of expected cell counts were below 5. The *t*‐test was employed for normally distributed continuous data, with Levene's test assessing variance equality. The Mann–Whitney *U*‐test was used for non‐normally distributed continuous variables. Analyses excluded missing data. All statistical tests were two‐sided, with significance set at *p*‐values <0.05.

Differences in LR and OS rates between LLN‐positive and LLN‐negative groups were assessed. Recurrence and survival data were displayed using the Kaplan–Meier curves and evaluated with Mantel–Cox logistic regression. Cox regression analysis was used to calculate hazard ratios (HR) at 3 and 5 years with confidence interval (CI) of 95%.

The analysis was adjusted for gender, age, clinical tumour stage, neoadjuvant therapy, ASA‐classification and tumour height. Confounding variables were determined through univariable analysis and constrained by the number of outcome events. Multivariable analysis used complete case analysis, excluding cases with missing data in any of the adjustment variables.

To evaluate difference in outcome for patients who underwent LLND, the same analyses described above were applied to LLN‐positive patients, with LLND used as the independent variable. The analysis was adjusted for the same confounding variables previously mentioned.

All statistical analyses were conducted using SPSS (IBM SPSS Statistics, version 29.0.2.0, IBM Corp., Armonk, NY, USA).

## RESULTS

**FIGURE 1:**
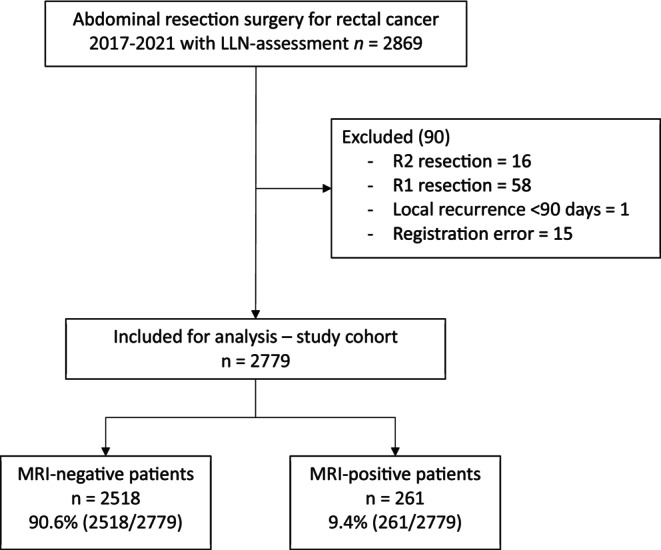
Study flow chart of patient inclusion and exclusion. LLN, lateral lymph node.

In all, 2869 patients diagnosed with rectal cancer were identified through the SCRCR. After excluding 90 patients with non‐radical resections (R1 and R2), early recurrences within 90 days of surgery and various registration errors, 2779 patients were analysed (Figure 1). Radiological assessments identified 90.6% (*n* = 2518/2779) of patients as having negative LLN and 9.4% (*n* = 261/2779) as having positive LLN.

Patients with positive LLN were younger and generally had more distally located tumours. However, in 17.2% of LLN‐positive patients, the tumour was located in the upper part of the rectum representing a rate of 5.8% (*n* = 45/774) MRI‐positive LLNs compared with 9.0% (*n* = 11/1229) and 13.9% (*n* = 103/760) in the middle and lower rectum, respectively. Among patients with LLN‐positive tumour in the upper rectum, 66.7% (*n* = 30/45) received neoadjuvant therapy, 13% (*n* = 6/45) underwent LLND and one (2.2%) patient developed a LR. Involvement of the mesorectal fascia and detection of positive mesorectal lymph nodes were more common in LLN‐positive patients, with a higher number of positive mesorectal lymph nodes overall (Table 1). LLN‐positive patients had more often suspected inguinal and para‐aortic lymph node metastases and histopathological findings including advanced tumour stage, tumour deposits, perineural infiltration and lymphovascular invasion (Table 2). However, LLN positivity was also detected in 53 patients with a histopathological tumour stage of T1 and T2.

**TABLE 1:** Baseline patient characteristics.

Patients	All patients	MRI‐negative	MRI‐positive	*p*‐value
	2779 (100)	2518 (90.6)	261 (9.4)	
Sex				
Male	1700 (61.2)	1516 (60.2)	184 (70.2)	0.001
Female	1079 (38.8)	1002 (39.8)	77 (29.8)	
Age				
Mean (±SD)	68.3 (11.2)	68.6 (11.3)	65.9 (10.8)	<0.001
BMI				
Mean (±SD)	26.1 (4.5)	26.1 (4.5)	26.3 (4.5)	0.480
Missing	29	28	1	
ASA class				
1	369 (13.3)	340 (13.5)	29 (11.1)	0.725
2	1585 (57.0)	1431 (56.8)	154 (59.0)	
3	731 (26.3)	663 (26.3)	68 (26.1)	
4	27 (1.0)	24 (1.0)	3 (1.1)	
Missing	67 (2.4)	60 (2.4)	7 (2.7)	
Tumour height (cm)				
Low (0–5)	760 (27.3)	657 (26.1)	103 (39.5)	<0.001
Middle (6–10)	1229 (44.9)	1118 (44.4)	111 (42.5)	
High (11–15)	774 (27.9)	729 (29.0)	45 (17.2)	
Missing	16 (0.5)	14 (0.6)	2 (0.8)	
cTNM stage				
1	644 (23.2)	644 (25.6)	0	<0.001
2	537 (19.3)	537 (21.3)	0	
3	1302 (46.9)	1094 (43.4)	208 (79.7)	
4	277 (10.0)	224 (8.9)	53 (20.3)	
Missing	19 (0.7)	19 (0.8)	0	
cT stage				
T1‐2	876 (31.5)	854 (33.9)	22 (8.4)	<0.001
T3‐4	1888 (67.9)	1649 (8.4)	239 (91.6)	
Missing	15 (0.5)	15 (0.6)	0	
Pelvic radiology				
MRI	2741 (98.6)	2480 (98.5)	261 (100)	0.460
PET‐CT	116 (4.2)	104 (4.1)	12 (4.6)	0.719
CT only	36 (1.3)	36 (1.4)	0	
MRF‐positive				
No	1556 (56.0)	1477 (58.7)	79 (30.3)	<0.001
Yes	442 (15.9)	353 (14.0)	89 (34.1)	
Missing	781 (28.1)	688 (27.3)	93 (35.6)	
Mesorectal LNs				
No	1251 (45.0)	1224 (48.6)	27 (10.3)	<0.001
Yes	1512 (54.4)	1279 (50.8)	233 (89.3)	
Missing	16 (0.6)	15 (0.6)	1 (0.4)	
Number of positive mesorectal LNs				
Median (IQR)	3 (2–5)	3 (2–5)	5 (3–8)	<0.001
N0/Missing	1284	1251	33	
Inguinal LNs				
No	1687 (60.7)	1570 (62.4)	117 (44.8)	<0.001
Yes	34 (1.2)	14 (0.6)	20 (7.7)	
Missing	1058 (38.1)	934 (37.1)	124 (47.5)	
Para‐aortic LNs				
No	1682 (60.5)	1556 (61.3)	126 (48.3)	<0.001
Yes	31 (1.1)	21 (0.8)	10 (3.8)	
Missing	1066 (38.4)	941 (37.4)	125 (47.9)	

Abbreviation: IQR, interquartile range; LNs, lymph nodes; MRF, mesorectal fascia; SD, standard deviation.

**TABLE 2:** Histopathology.

Patients	All patients	MRI‐negative	MRI‐positive	*p*‐value
	2779 (100)	2518 (90.6)	261 (9.4)	
Tumour stage (final)				
1	705 (25.4)	682 (27.1)	23 (8.8)	<0.001
2	592 (21.3)	562 (22.3)	30 (11.5)	
3	1182 (42.5)	1038 (41.2)	144 (55.2)	
4	297 (10.7)	233 (9.3)	64 (24.5)	
Missing	3 (0.1)	3 (0.1)	0	
(y)pT stage				
T0	71 (2.6)	54 (2.1)	17 (6.5)	<0.001
T1‐2	1086 (39.1)	1022 (40.6)	64 (24.5)	
T3‐4	1574 (56.6)	1402 (55.7)	172 (65.9)	
Missing	48 (1.7)	40 (1.6)	8 (3.1)	
(y)pN stage				
N0	1690 (60.8)	1559 (61.9)	131 (50.2)	<0.001
N1	803 (28.9)	711 (28.2)	92 (35.2)	
N2	247 (8.9)	214 (8.5)	33 (12.6)	
Missing	39 (1.4)	34 (1.4)	5 (1.9)	
Tumour deposits				
No	2215 (79.7)	2029 (80.6)	186 (71.3)	<0.001
Yes	474 (17.1)	408 (16.2)	66 (25.3)	
Missing	90 (3.2)	81 (3.2)	9 (3.4)	
Number of tumour deposits				
Median ± IQR	2 (1–3)	2 (1–3)	2 (1–4)	0.228
Missing	16			
Perineural infiltration				
No	2089 (75.2)	1912 (75.9)	177 (67.8)	0.009
Yes	611 (22.0)	538 (21.4)	73 (28.0)	
Missing	79 (2.8)	68 (2.7)	11 (4.2)	
Vascular invasion				
No	1836 (66.1)	1685 (66.9)	151 (57.9)	0.005
Yes	854 (30.7)	755 (30.0)	99 (37.9)	
Missing	89 (3.2)	78 (3.1)	11 (4.2)	
Complete response (ypT0N0)				
No	2674 (96.2)	2434 (96.7)	240 (92.0)	<0.001
Yes	58 (2.1)	44 (1.7)	14 (5.4)	
Missing	47 (1.7)	40 (1.6)	7 (2.4)	

Abbreviation: IQR, interquartile range.

The majority of LLN‐positive patients received neoadjuvant CRT with subsequent re‐evaluation. Of patients with initially suspected LLNM, 18.4% (*n* = 48/261) underwent LLND (Table 3). Patients with stage 4 disease, including inguinal and para‐aortic node involvement, were more frequently treated with neoadjuvant therapy including chemotherapy (Stage 1: 0.5%, Stage 2: 13.4%, Stage 3: 28.6%, Stage 4: 54,8%). Patients with primarily resectable disease may have received short course radiotherapy and surgical resection of metastases, in accordance with national guidelines at the time.

**TABLE 3:** Therapy.

Patients	All patients	MRI‐negative	MRI‐positive	*p*‐value
	2779 (100)	2518 (90.6)	261 (9.4)	
Neoadjuvant therapy				
None	1137 (40.9)	1099 (43.6)	38 (14.6)	<0.001
RT	1037 (37.3)	963 (38.2)	74 (28.4)	
CRT	555 (20.0)	413 (16.4)	142 (54.4)	
CHT	46 (1.7)	39 (1.5)	7 (2.7)	
Missing	4 (0.1)	4 (0.2)	0	
Surgical resection				
AR	1290 (46.4)	1206 (47.9)	84 (32.2)	<0.001
APR	1065 (38.3)	923 (36.7)	142 (54.4)	
Hartmann	424 (15.3)	389 (15.4)	35 (13.4)	
LLND				
No	2714 (97.7)	2501 (99.3)	213 (81.6)	<0.001
Yes	65 (2.3)	17 (0.7)	48 (18.4)	
Missing	0	0	0	

Abbreviations: APR, abdominoperineal resection; AR, anterior resection; CHT, chemotherapy; CRT, chemoradiotherapy; LLND, lateral lymph node dissection; RT, radiotherapy.

An additional analysis was conducted on a small regional subgroup (*n* = 20) who had undergone LLND. Histopathological examination revealed a 25% prevalence of LLNM in the resected specimens.

### Local recurrence

**FIGURE 2:**
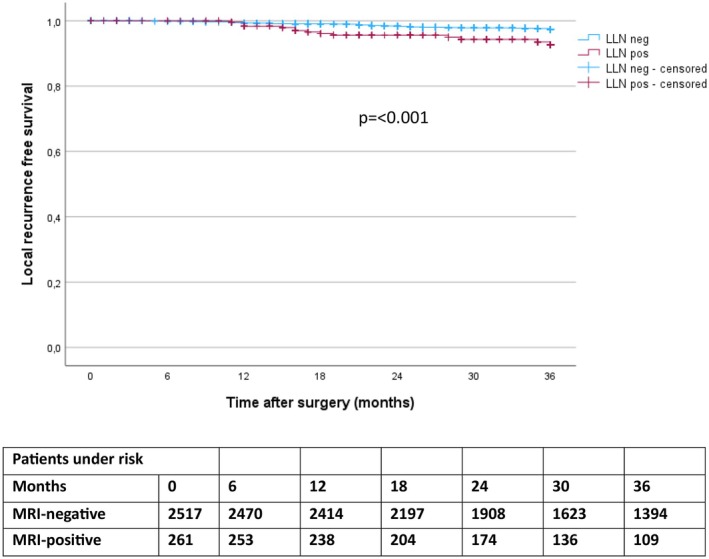
Kaplan–Meier curve of local recurrence‐free survival over 3 years after surgical resection. LLN, lateral lymph node.

LR was diagnosed within 3 years in 68 patients; 2.1% (*n* = 54/2518) in LLN‐negative patients and 5.4% (*n* = 14/261) in LLN‐positive patients. Univariable Cox regression analysis indicated an increased risk of LR in LLN‐positive patients after 3 years (HR 2.79 (CI 1.55–5.03); *p* < 0.001) (Figure 2 and Table 4). The difference withstood in the multivariable analysis (HR 1.97 (CI 1.04–3.73); *p* 0.037).

**TABLE 4:** Cox regression analysis of local recurrence at 3 years.

	*n*	HR (CI 95%)	*p*‐value
3‐year local recurrence—univariable			
MRI neg	2518	1.00	ref.
MRI pos	261	2.79 (1.55–5.03)	<0.001
3‐year local recurrence after surgery—multivariable^a^			
MRI neg	2483	1.00	ref.
MRI pos	259	1.97 (1.04–3.73)	0.037
Adjustments in multivariable analysis			
Gender		1.06 (0.65–1.75)	0.812
Age		0.98 (0.96–1.01)	0.132
Stage II		1.36 (0.54–3.38)	0.514
Stage III		1.48 (0.65–3.38)	0.350
Stage IV		3.91 (1.56–9.81)	0.004
ASA 2		1.72 (0.72–4.13)	0.224
ASA 3		2.21 (0.85–5.78)	0.104
ASA 4		n/a	n/a
Medium (6–10)		2.17 (0.98–4.80)	0.057
Low (0–5)		2.30 (1.12–4.70)	0.023
NeoAdj		0.94 (0.50–1.78)	0.856

^a^ Multivariable analysis adjusted for gender, age, cStage, neoadjuvant therapy, ASA class and tumour height (cm).

Among LLN‐positive patients, in the non‐LLND subgroup LR was diagnosed in 3.8% (*n* = 8/213) and 4.2% (*n* = 9/213) within 3 and 5 years, respectively. In LLN‐positive patients who underwent LLND, LR was diagnosed in 12.5% (*n* = 6/48) within 3 and 5 years. Subsequently, LLND was associated with increased LR risk at 3 years (HR 3.73 (CI 1.93–10.76); *p* 0.015) as well as at 5 years (HR 3.36 (CI 1.19–9.44); *p* 0.022) in univariable analysis. In multivariable analysis, the difference did not remain neither at 3 years (HR 2.31 (CI 0.72–7.46); *p* 0.160) nor after 5 years (HR 2.22 (CI 0.71–6.93); *p* 0.171). Univariable analysis was performed for each of the adjustment variables separately to identify confounding effects in this subgroup. The increased risk of LR remained associated with LLND and tumour stage in these analyses, however, not with the other possible confounders.

### Overall survival

**FIGURE 3:**
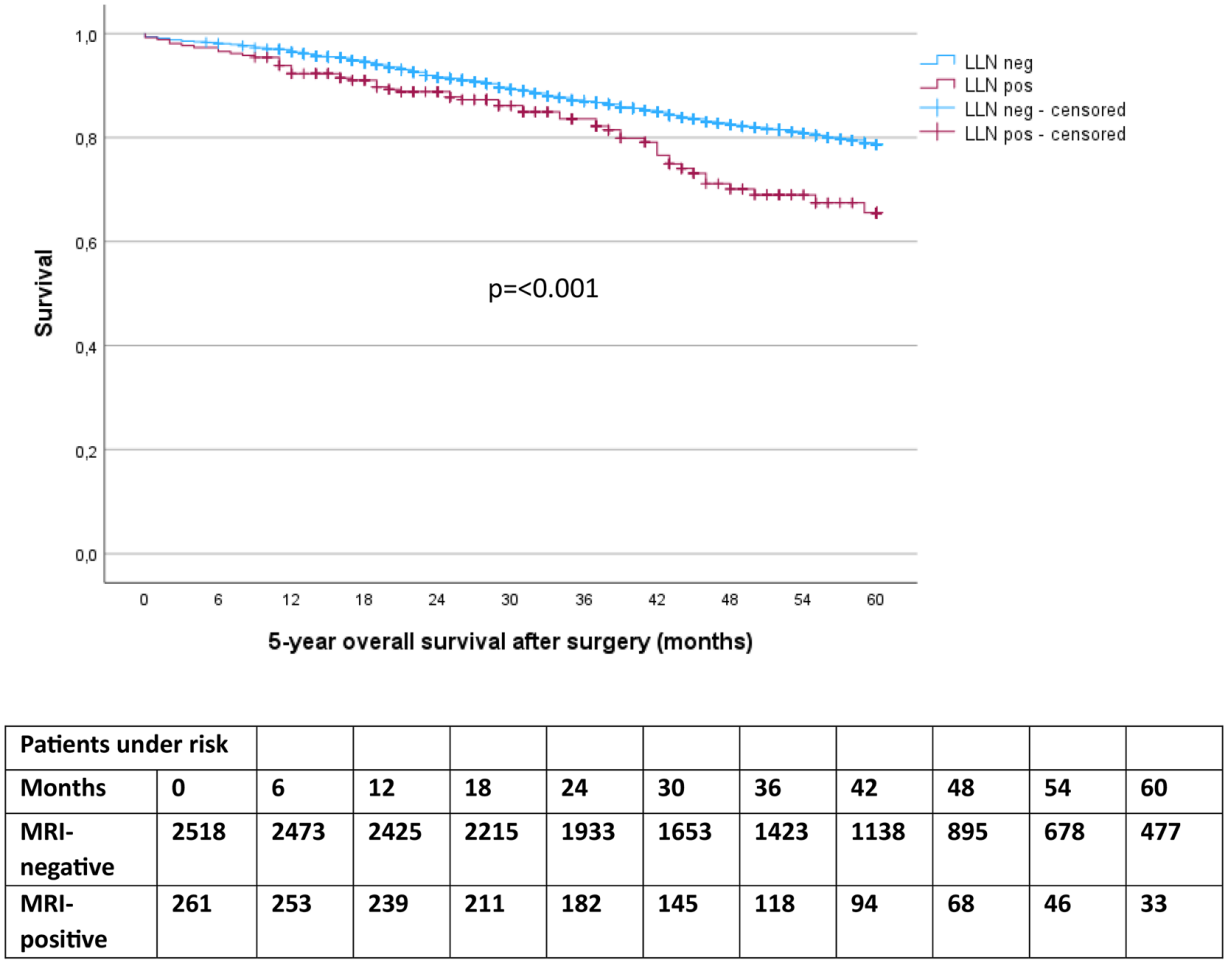
Kaplan–Meier curve of overall survival over 5 years after surgical resection. LLN, lateral lymph node.

At 3 years after surgery, 11.3% (*n* = 285/2518) of LLN‐negative and 14.2% (*n* = 37/261) of LLN‐positive patients had died; log‐rank (*p* = 0.078) and univariable Cox regression analysis (*p* = 0.080) (Table 5). After 5 years, mortality had increased to 14.9% (*n* = 375/2518) in LLN‐negative and 21.5% (*n* = 56/261) in LLN‐positive patients; log‐rank (*p* ≤ 0.001) and univariable Cox regression analysis (*p* ≤ 0.001) (Figure 3 and Table 5).

**TABLE 5:** Cox regression analysis of overall survival at 3 and 5 years.

	*n*	HR (CI 95%)	*p*‐value
3‐year overall survival after surgery—univariable			
MRI neg	2518	1.00	ref.
MRI pos	261	1.36 (0.96–1.91)	0.080
3‐year overall survival after surgery—multivariable^a^			
MRI neg	2435	1.00	ref.
MRI pos	254	1.06 (0.73–1.54)	0.764
Adjustments in multivariable analysis			
Gender		0.932 (0.74–1.18)	0.555
Age		1.05 (1.03–1.06)	<0.001
Stage II		1.25 (0.84–1.88)	0.276
Stage III		1.62 (1.12–2.34)	0.011
Stage IV		4.13 (2.74–6.24)	<0.001
ASA 2		1.29 (0.79–2.09)	0.310
ASA 3		2.19 (1.33–3.61)	0.002
ASA 4		4.57 (2.03–10.3)	<0.001
Medium (6–10)		1.42 (1.04–1.95)	0.027
Low (0–5)		0.94 (0.70–1.25)	0.650
NeoAdj		1.05 (0.79–1.39)	0.752
5‐year overall survival after surgery—univariable			
MRI neg	2518	1.00	ref.
MRI pos	261	1.64 (1.24–2.17)	<0.001
5‐year overall survival after surgery—multivariable^a^			
MRI neg	2435	1.00	ref.
MRI pos	254	1.32 (0.97–1.80)	0.075
Adjustments in multivariable analysis			
Gender		0.83 (0.69–1.03)	0.084
Age		1.05 (1.04–1.06)	<0.001
Stage II		1.33 (0.95–1.87)	0.098
Stage III		1.64 (1.20–2.24)	0.002
Stage IV		3.86 (2.69–5.53)	<0.001
ASA 2		1.32 (0.88–1.98)	0.181
ASA 3		2.14 (1.40–3.25)	<0.001
ASA 4		4.50 (2.20–9.20)	<0.001
Medium (6–10)		1.53 (1.17–2.02)	0.002
Low (0–5)		0.99 (0.77–1.28)	0.956
NeoAdj		1.01 (076–1.29)	0.963

^a^ Multivariable analysis adjusted for gender, age, cStage, neoadjuvant therapy, ASA class and tumour height (cm).

In multivariable Cox regression analysis, no difference in neither 3 nor 5‐year OS was observed as shown in Table 5.

There was no difference in OS at 3 years for patients who underwent LLND in univariable (HR 0.92 (CI 0.41–2.10); *p* 0.847) or multivariable analysis (HR 1.34 (CI 0.55–3.30); *p* 0.518). Similarly, OS remained unaffected by LLND among LLN‐positive patients after 5 years in univariable (HR 0.80 (CI 0.42–1.56); *p* 0.516) and multivariable analysis (HR 0.97 (CI 0.48–2.00); *p* 0.942). The actual survival rate in the LLN‐positive patients at 5 years was 78.9% (*n* = 168/213) in the non‐LLND subgroup versus 77.1% (*n* = 37/48) among LLND patients.

## DISCUSSION AND CONCLUSIONS

In this study, 9.8% of 2779 patients with rectal cancer diagnosed between 2017 and 2021 were LLN‐positive based on MRI staging. Factors associated with LLN positivity were male gender, younger age, lower tumour height, advanced tumour stage as well as inguinal and para‐aortic lymph node involvement. LLN‐positive patients more often received CRT and had an increased 3‐year LR and reduced OS in univariable analysis. LLND appeared to decrease LR risk in the univariable analysis without affecting mortality. Notably, 17.2% of LLN‐positive tumours were located in the upper part of the rectum and were also identified in T1‐2 cases, warranting awareness.

The foremost strength of this study is its large nationwide cohort. To our knowledge, this study is the first to make use of the SCRCR variable for LLN‐positivity. Limitations include the retrospective design of the study. Furthermore, radiology and interpretation were performed within regular clinical routine and were not subjected to centralized review. Information concerning the re‐evaluating MRI was not accessible through the registry, nor so how LLND had been performed. The methods of LLND were not standardized, and it is not clear if the patients with LLND underwent complete LLND uni‐ or bilaterally or if only dissection of the suspected LLN was undertaken (i.e. node‐picking). Also, data on adjuvant therapy were not available, which may have affected outcome measures.

### Prevalence

Reported LLN positivity varies widely, presumably reflecting differences in study populations and methodologies. In a multicentre analysis of Eastern and Western cohorts, Ogura et al. found a pooled prevalence of MRI‐detected malignant LLNs at 17.1% [14]. Higher LLN‐positivity rates like this are often reported in studies that exclude high rectal tumours, whereas the present study included tumours of all heights. Takahashi et al. found LLNM in 8.6% of all rectal cancers and 16.4% of low rectal cancers, corresponding to our findings [22].

Another possible explanation for the lower LLN positivity in this study may be classification differences. In Sweden, lymph nodes along the internal iliac and obturator arteries constitute N‐disease, while those along the external iliac and common iliac arteries are considered metastases following the AJCC 8th edition [18, 23]. By contrast, Japanese guidelines include external iliac and common iliac nodes as LLNs, potentially leading to underestimation in the Swedish data [24]. A meta‐analysis of 31 Asian studies reported a pooled prevalence of histopathologically confirmed LLNM at 17.3% [25]. LLNM in resected specimens may be higher in countries where LLND is the preferred primary treatment where LLNs are not treated with CRT prior to surgery. LLNM is more common in patients with persistent LLN positivity after CRT compared to those with a treatment response [26]. A study limitation was the lack of data on treatment response following neoadjuvant therapy, as well as the specific reasons for performing or omitting LLND in individual patients. In addition to radiological response, other factors such as overall disease progression, patient health status or patient preference might have influenced whether LLND was ultimately performed.

Ogura et al. found that 18% of LLNs remained suspicious post‐CRT, using a 7 mm short‐axis cutoff, similar to this study's LLND rate of 17.2%, suggesting that LLND cases likely represent LLNs unresponsive to neoadjuvant therapy [14]. A few LLND were performed, despite LLN‐negativity, possibly due to differences in radiological definitions of metastatic disease in SCRCR. Due to limitations in medical chart availability, only patients treated in a geographic sub‐region could be investigated for histopathological prevalence of LLNM after resection. Among this regional subgroup of LLND patients in this study, most of whom received CRT, only 25% had LLNM according to the histopathological analysis. This is lower than those reported by Peacock et al. (34.1%; 32.5%) [16, 27].

### Factors associated with MRI‐detected LLN positivity

In this study, lower age was a risk factor for LLN positivity, consistent with a systematic review by Gulevski et al. and a meta‐analysis by Zeng et al. [8, 28] By contrast, the higher risk of LLN‐positivity in women reported in these studies was not verified. The discrepancy may be due to male overrepresentation in the LLN‐positive group in some studies, though not statistically significant.

LLNM, confirmed with histopathology, is generally associated with lower tumour location, in accordance with the current study [5]. However, 17.2% of LLN‐positive tumours were positioned 11 to 15 cm from the anal verge, an important finding since many studies focus on low rectal tumours when evaluating LLN involvement. The true incidence of LLNM in upper rectal cancers is unknown and the rate of 5.8% LLN‐positive patients in the current study might reflect an overestimation which could have induced overtreatment. In the current study, there was no evident increase in LR rate among patients with LLN‐positive cancer in the upper rectum.

In the current study, factors associated with more advanced tumours, such as mesorectal fascia involvement, perineural and vascular invasion and mesorectal LN metastases were more frequent in patients with LLN positivity. However, LLN with malignant features was not exclusive to patients with advanced‐stage rectal cancer; close to ten percent of the LLN‐positive group had a histopathological tumour stage of T1 and T2.

### Local recurrence and overall survival

This study indicates that MRI‐detected LLN positivity is associated with a higher risk of LR after 3 years, also when adjusting for other risk factors. In univariable analysis, LLND by any method seemed to be associated with an increased risk of LR, but this effect was not statistically significant when adjusted for clinical tumour stage. No correlation between LLND and overall survival was found. The lack of standardization regarding LLND in the cohort has to be taken into account when interpreting these findings. Additionally, information on the location of LR within the pelvis is not available in registry follow‐up data but would have been of interest since most, but not all, LR occurs in lateral compartments.

There are reports of patients with suspected LLNM having responded well to neoadjuvant CRT based on follow‐up imaging but still had positive LLNM upon pathological examination [29]. This indicates a potential benefit of LLND; however, some patients still experience LR despite undergoing the procedure [30].

The true incidence of LLNM in the current study is uncertain due to the absence of chart review, the lack of standardization in LLND technique and the lack of histopathological evaluation of MRI‐positive lymph nodes prior to neoadjuvant therapy and surgery. Additionally, direct comparison with Japanese studies is limited by differences in tumour height, indications for neoadjuvant therapy and the lack of histopathological confirmation of LLNM [2]. LLNM was found in 25% of patients undergoing LLND, indicating possible overestimation of LLNM preoperatively. However, exclusion of patients with good radiological response to neoadjuvant therapy may also have led to underestimation.

Patients who ultimately underwent LLND in accordance with the treatment guidelines would have had both primarily MRI‐positive LLNM and persisting nodes after neoadjuvant therapy. The observed association between LLND and recurrence in LLN‐positive patients likely reflects the increased baseline risk, and no conclusions can be drawn about the effect of LLND on this outcome due to the risk of selection bias. Furthermore, it cannot be ruled out that the lack of standardization and centralization of LLND surgery during the study period might have affected the outcomes. Several retrospective studies show mixed results. Some suggest that both the Western CRT + TME and Eastern TME + LLND approaches are similarly efficient in reducing LR when compared to TME alone [3, 31, 32]. A randomized controlled study by Fujita et al. (*n* = 701) showed that adding LLND to TME, without neoadjuvant therapy, reduced LRs [15]. Another RCT (*n* = 51) found no difference in LR rates or survival between patients receiving neoadjuvant RT followed by TME + LLND or TME alone [33, 34]. Neither of these RCTs included patients with preoperative LLN positivity, but LLND was performed prophylactically in high‐risk patients. A meta‐analysis found no reduction in LR with the addition of LLND to TME surgery [35]. In the current study, OS was lower after 5 years in patients with MRI‐detected LLN positivity in the univariate analyses, but not in multivariable analyses. This suggests that LLNM may correlate with other risk factors indicative of more advanced disease, and how LLNM itself affects OS or can be improved by different treatment strategies remains unclear. The current study was also limited by the absence of total neoadjuvant therapeutic strategies, which have been increasingly adopted.

### Future perspectives

The true prevalence and optimal treatment of LLNM remain elusive due to non‐standardized diagnostics and variable treatment outcomes. Ongoing studies, such as the LaNoReC study by the Dutch Colorectal cancer group and the forthcoming LATITUDE study conducted by the Scandinavian Surgical Outcomes Research Group, aim to clarify indications for LLND and support standardized treatment strategies [36]. Future efforts, including targeted chart reviews to enable more detailed analysis of local recurrence patterns, including type and location, are needed to better characterize recurrence patterns and identify which patients truly benefit from more extensive surgery.

## AUTHOR CONTRIBUTIONS

**Erik Agger:** Conceptualization; data curation; formal analysis; visualization; writing – original draft; methodology; investigation; project administration; writing – review and editing. **Cecilia Dahlbäck:** Conceptualization; formal analysis; writing – original draft; methodology; writing – review and editing. **Cedric Delorme:** Data curation; formal analysis; writing – original draft; investigation. **Pamela Buchwald:** Conceptualization; data curation; formal analysis; writing – original draft; methodology; project administration; writing – review and editing.

## FUNDING INFORMATION

This study was funded by ALF‐project grants.

## CONFLICT OF INTEREST STATEMENT

The authors declare no conflict of interest.

## ETHICS STATEMENT

The study was approved by the Ethics Review Board (Dnr: 2019–02175 with supplements Dnr: 2023–00372‐02 and Dnr: 2023–04669‐02).

## PATIENT CONSENT STATEMENT

All patients registered in the Swedish ColoRectal Cancer Registry provide informed consent before being included. Patients can at any time withdraw their consent and have all health data permanently removed.

## CLINICAL TRIAL REGISTRATION

The trial has not been pre‐registered.

## Supporting information


Appendix S1.

## Data Availability

The data that support the findings of this study are available from SCRCR. Restrictions apply to the availability of these data, which were used under licence for this study. Data are available from the authors with the permission of SCRCR and the Ethics Review Board.
